# Biofilm and surface-motility profiles under polymyxin B stress in multidrug-resistant KAPE pathogens isolated from Ghanaian hospital ICUs

**DOI:** 10.3389/ebm.2025.10350

**Published:** 2025-06-06

**Authors:** Molly K. Abban, Eunice Ampadubea Ayerakwa, Abiola Isawumi

**Affiliations:** ^1^ West African Centre for Cell Biology of Infectious Pathogens, College of Basic and Applied Sciences, University of Ghana, Accra, Ghana; ^2^ Department of Biochemistry, Cell and Molecular Biology, College of Basic and Applied Sciences, University of Ghana, Accra, Ghana

**Keywords:** KAPE pathogens, polymyxin, biofilms, surface-motility, swarming

## Abstract

The threat of antimicrobial resistance in Ghana is increasing with the recent emergence of KAPE pathogens (*K. pneumoniae*, *A. baumannii*, *P. aeruginosa* and *Enterobacter* species) from the hospital environment. As opportunistic pathogens, KAPE leverage the formation of biofilms and swarms to survive stringent environmental conditions. As research continues to investigate approaches that bacteria employ to exacerbate infection, this study explored biofilm and swarm formation in MDR KAPE pathogens under polymyxin B stress emerging from Ghanaian hospitals. The antimicrobial susceptibility profile of KAPE pathogens to conventional antibiotics and polymyxin B was investigated via antibiotic disk diffusion and broth microdilution assays. Biofilm inhibition and eradication assays, swarm motility and a resazurin-based metabolic assay were used to profile bacterial phenotypic characteristics under polymyxin B stress. The strains exhibited resistance to the tested antibiotics with a high level of resistance to polymyxin B (PMB) (≥512 μg/mL). Additionally, the strains formed biofilms and bacterial swarms at 37°C. In the presence of PMB (≥512 μg/mL), KAPE pathogens formed swarms with no significant reduction in bacterial swarms at 1,048 μg/mL. Biofilm was observed for all strains with PMB neither inhibiting nor eradicating at high PMB (2048 μg/mL). Additionally, there were no significant differences in the phenotypic and antimicrobial susceptibility profiles of clinical and environmental KAPE pathogens from Ghanaian ICUs. Overall, the study established that clinical and environmental KAPE pathogens from Ghanaian ICUs exhibit adaptive phenotypic and resistance characteristics that could potentially enhance bacterial survival during host colonization and infection. This could undermine treatment strategies and pose public health challenges in Ghana.

## Impact statement

Critical priority bacterial pathogens pose serious public health challenges with increasing therapeutic failures. This is attributed to the development of survival mechanisms that facilitate antimicrobial resistance, host colonization and immune evasion. These mechanisms depend on the expression of diverse phenotypic traits including biofilm formation and surface-motility. Bacteria use biofilm and motility machinery to survive stringent environmental conditions, initiate virulence and persist in the presence of antibiotics. These traits contribute to the pathogenicity of bacteria and increase the burden of antimicrobial-resistant infections. This study examined biofilm-motility interplay as a mechanism of tolerance to polymyxin B.

## Introduction

The hospital environment represents a model of microbial interaction because it plays a key role in disease pathogenesis. The interplay between humans and bacteria in the hospital environment threatens the safety of all hospital users, thereby increasing the frequency of hospital-acquired infections (HAIs) [1–3]. There is increased morbidity and mortality associated with HAIs [4] with ventilator-associated pneumonia (VAP), central line-associated bloodstream infections (CLABSI), catheter-associated urinary tract infections (CAUTI) and surgical site infections (SSI) being the predominant HAIs [5–7]. Incidence rates of HAIs (5.7%–19.1%) have been reported in developing countries including Ghana (8.2%) [3] with VAP, CLABSI and CAUTI being frequently reported [6–8]. Bacteria are the most commonly isolated pathogens and contribute to 87% of reported HAIs [5, 7, 9]. They survive in the hospital largely as commensals and opportunistic pathogens from normal human flora, immunocompromised patients and the general hospital environment. Globally, reported bacterial strains implicated in HAIs include *Pseudomonas aeruginosa, Acinetobacter baumannii*, members of the *Enterobacteriaceae* and *Staphylococcus aureus* [7, 10–12]. Of this group, Gram-negative bacteria represent a high risk to public health due to an increase in AMR [1, 7].

Gram-negative KAPE (*K. pneumoniae*, *A. baumannii*, *P. aeruginosa* and *Enterobacter* spp.) [1] or the friendly amendment ESCAPE [13] (*C. difficile*, *A. baumannii*, *P. aeruginosa* and Enterobacteriaceae), have been implicated in major bacterial infections and described as extremely critical with global precedence [14, 15]. They are ubiquitous and primarily associated with HAIs particularly among immunocompromised and critically ill patients [1, 6]. They have a tendency to circumvent lethal doses of antibiotics. These pathogens present with multidrug resistant, extensively drug-resistant or pan-drug-resistant phenotypes [16, 17] and infections resulting from these resistant Gram-negative pathogens have been associated with poorer patient outcomes than susceptible isolates [18]. The mechanisms employed by KAPE pathogens to display resistance and induce virulence include drug inactivation, modification of the target site, and reduction in drug permeability [19] and quorum sensing by utilizing surface-motility and biofilm development to promote resistant populations [20].

Swarming surface-motility and biofilm formation are hallmark survival mechanisms utilized by multidrug-resistant pathogens [21] during harsh, unfavorable conditions such as antibiotic treatment. Both processes allow for rapid colonization and establishment of infection with bacterial swarming enabling initial attachment of cells to surfaces including catheters to induce biofilm formation [22, 23]. These multicellular adaptations provide strains with mechanical and biochemical advantages, making it difficult to eliminate bacteria using conventional antibiotics [24]. In addition, swarming and biofilm-forming cells exhibit increased adaptive phenotypic resistance and tolerance due to innate and acquired resistance markers that promote AMR [25, 26]. Increased tolerance and resistance to conventional antibiotics lead to dependence on last-resort antibiotics such as carbapenems and polymyxins [21]. For antibiotics to disrupt and inhibit swarming and biofilm formation, higher antibiotic concentrations, combinations, or disruption of gene targets are required [26]. Although some studies report an inverse relationship between biofilm formation and swarm motility [27], the ability to form these coordinated multicellular behaviors particularly in MDR strains leads to increased virulence and pathogenicity [28–30].

There are few studies on how biofilm and motility, as phenotypic factors contribute to HAIs in Gram-negative bacteria in Ghana. Also, how these factors enhance the level of AMR, leading to reduced treatment options in the Ghanaian hospital setting, has not been fully explored. The majority of pathogens implicated in HAIs exhibit a tendency to colonize diverse surfaces via the formation of biofilms [29, 31] and surface swarm motility [21, 24]. In this study, we explored the phenotypic characteristics of clinical and environmental Gram-negative KAPE, *Citrobacter* sp. and *E. coli* from ICUs of Ghanaian hospitals. Additionally, the interplay of surface-motility and biofilm profiles under polymyxin B as survival characteristics was explored.

## Materials and methods

### Bacterial strains and culture conditions

Archived Gram-negative bacterial strains of KAPE pathogens (obtained from air, fomites, and patients) from the ABISA™ bacterial culture library at the Department of Biochemistry, Cell and Molecular Biology, University of Ghana were used in this study (This study is part of a larger study approved by the Ghana Health Service: GHS-ERC01/02/17). Six environmental and six clinical strains associated with HAIs (*Klebsiella pneumoniae, Acinetobacter baumannii*, *Enterobacter* sp., *Citrobacter* sp. and *Pseudomonas aeruginosa*) were selected. Control strains were UK19 *E. coli* (ATCC 25922) for antimicrobial susceptibility testing, *Pseudomonas aeruginosa* (PS03) for biofilms and *Proteus mirabilis* (PT01) for swarm motility. Bacterial strains were recovered from a −80°C freezer and revived in Luria-Bertani broth (LB) (Invitrogen Life Tech, United States) at 37°C for 18 h with shaking at 60 rpm. Strains were refreshed in LB broth, streaked on MacConkey agar (Oxoid, England, CM0007B) and incubated at 37°C overnight.

### AMR susceptibility profiles of strains

Fifteen standard commercial antibiotics including cloxacillin (5 µg), nitrofurantoin (200 µg), penicillin (15 µg), ampicillin (10 µg), nalidixic-acid (30 µg), ceftazidime (30 µg), chloramphenicol (50 µg), cefotaxime (10 µg), cefuroxime (30 µg), cotrimoxazole (25 µg), gentamycin (10 µg), tetracycline (30 µg), ceftriaxone (30 µg), erythromycin (15 µg) and flucloxacillin (10 µg) were used. Briefly, overnight bacterial culture was adjusted to 0.5 McFarland, seeded on sterile Mueller Hinton agar (Invitrogen life tech) plates and antibiotic discs were aseptically applied (incubation, 16–18 h at 37°C). The diameters of the zone of inhibition were recorded to the nearest millimeter (mm) and strains were classified as resistant, intermediate, or susceptible based on CLSI guidelines [32, 33]. The broth microdilution assay with polymyxin B (PMB) was conducted as previously described [34]. Briefly, PMB powder was prepared to a stock concentration of 12,000 μg/mL. Broth microdilution was performed with cation-adjusted Mueller Hinton broth in a range of two-fold dilutions (0.16–2,048 μg/mL) of PMB. One hundred microliters of PMB were transferred to 96-well plates and a final bacterial inoculum of 100 µL (1–5 × 10^5^ CFU/mL) was transferred to each well. The plates were incubated with shaking at 37°C for 18 h and the absorbance was read with a multimode microplate reader (Varioskan LUX Thermo Fisher Scientific). The minimum inhibitory concentration (MIC) was calculated as the percentage of OD < 10.

### Bacterial surface-motility assay

The swarming motility assay was performed as described by Morales-Soto et al. [35] with a few modifications, with and without PMB. Nutrient agar (Oxoid) was prepared to a concentration of 0.5% (w/v). The media was cooled to 60°C, and 15 mL was transferred to 60 mm Petri dishes. The plates were left to air dry for 1 h. Each strain was cultured to log phase (OD_600_ 0.2–0.5∼1 × 10^5-6^), harvested (5,000 rpm/5 min), and resuspended in double-distilled water. Five microliters of culture were spotted in the center of swarm media plates and incubated at 25°C, 37°C and 45°C for 24–72 h. Motility was assessed by measuring the diameter (mm) of the widest point of spread. For the swarm assay with PMB, nutrient agar plates were seeded with 512 μg/mL, 1,024 μg/mL and 2048 μg/mL PMB. Five microliters of bacterial culture at the log phase (OD_600_ 0.2–0.5), was spotted onto the center of the plates (25°C, 37°C, 45°C for 24–72 h). Biological and technical replicates were performed for each strain.

### Biofilm assays

Biofilm formation was assayed with the capillary tube adherence method and the 96-well microtiter plates (MTP) adapted from O’Toole, 2011 [34]. Briefly, 200 μL and 2 mL overnight cultures normalized to OD_600_ 0.1 in LB broth were transferred to 96-well plates and capillary tubes respectively and incubated for 3–5 days at 37°C. Spent media were removed and the plates/tubes were washed three times with sterile distilled water to remove loosely adherent bacteria. Plates/tubes were air-dried for 30 min, stained with 0.1% (w/v) crystal violet solution, and incubated at room temperature for 30 min. Plates were washed with sterile distilled water, air-dried and quantitatively assessed with 200 µL of 96% (v/v) ethanol and absorbance was determined at 590 nm. The data were interpreted according to the cut-off value (ODc) adapted from Stepanović et al., [36]. The isolates were characterized as no biofilm producers when OD ≤ ODc, weak when ODc < OD ≤ 2ODc, moderate with 2ODc < OD ≤ 4ODc, and strong with OD > 4ODc, where OD represents the absorbance value. For the biofilm inhibition assay, the MIC established for the strains was used as the standard condition to determine the biofilm inhibitory concentration. Briefly, microtiter wells were seeded with 100 µL of standardized culture at log-phase (OD_600_ 0.2–0.5). 100 μl of PMB at 512 μg/mL, 1,024 μg/mL and 2048 μg/mL were transferred to the wells and incubated at 37°C for 5 days. The wells were washed and subjected to crystal violet staining to quantify biofilm products, and bacterial viability was confirmed with resazurin assay [38]. The Biofilm eradication assay was performed as previously described [38]. Preformed biofilms were treated with 200 µL of PMB at 512 μg/mL, 1,024 μg/mL and 2048 μg/mL. Plates were incubated for 18–24 h at 37°C. Crystal violet and resazurin were used to quantify biofilm formation and determine the level of bacterial viability [38].

### Statistical analysis

The data were expressed as mean ± standard deviation and analyzed using Microsoft Office Excel and GraphPad Prism 7.0 (GraphPad Software, Inc. CA, USA). One-way ANOVA and Dunnett’s correction test were used to compare means between biofilm formation in strains relative to their untreated controls. Two-way ANOVA and Dunnett’s multiple comparison test were used to compare means between swarming strains under the different treatment conditions and incubation times. For the metabolic assay, two-way ANOVA and Dunnett’s multiple comparison were employed to compare controls (*Pseudomonas* sp. (PS03) and Negative control (PC)) to test strains. P < 0.05, statistically significant; ns (P > 0.05); *P < 0.05, **P < 0.009, ***P = 0.001, ****P < 0.0001.

## Results

### Strains are multidrug resistant with high levels of resistance to last-resort antibiotics

**FIGURE 1:**
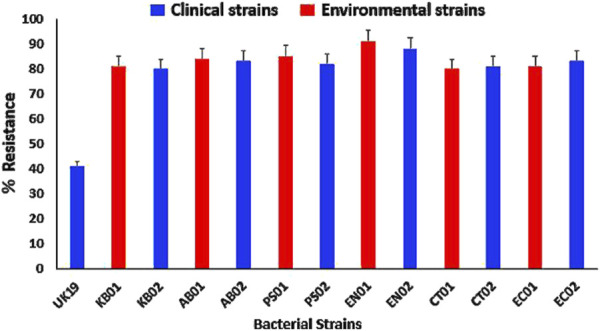
AMR profiles to conventional antibiotics relative to the UK19 *E. coli* control strain. (KB01, KB02) *K. pneumoniae*; (AB01, AB02) *A. baumannii*; (PS01, PS02) *P. aeruginosa*; (EN01, EN02) *Enterobacter* sp.; (CT01, CT02) *C. freundii*; (EC01, EC02) *E. coli*. Error bars indicate the percentage of resistance of the strains to the tested antibiotics.

The strains were tested against 15 different antibiotics belonging to 8 classes (β-lactams, macrolides, aminoglycosides, nitrofurans, sulfonamides, phenicols, tetracyclines and quinolones). All the strains were highly resistant with at least 80% levels of AMR to the tested antibiotics (Figure 1). The strains were resistant to at least two of the eight classes of antibiotics, with different resistance patterns and a multiple antibiotic resistance index of ≥0.8 relative to 0.4 for the *E. coli* control strain (Supplementary) indicating high levels of resistance per the CLSI guidelines. All strains displayed high levels of resistance to PMB with a MIC of 512 μg/mL, which was above the CLSI breakpoint for resistance of ≤4 μg/mL (Supplementary).

### Strains have strong biofilm phenotypes and swarm at high polymyxin B concentrations

**FIGURE 2:**
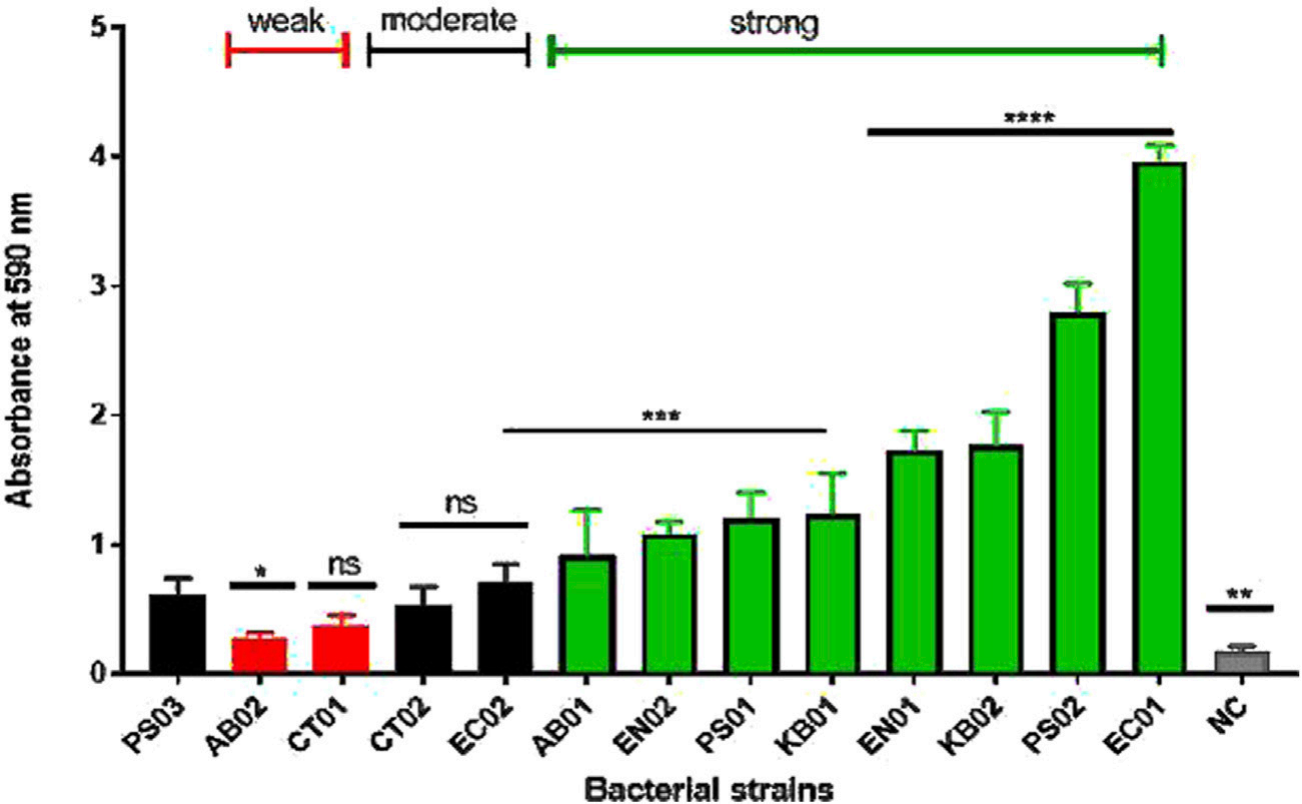
Biofilm profiles of clinical and environmental strains biofilm biomass was measured at 590 nm after 0.1% crystal violet staining. The relative biofilm produced for the strains was compared to PS03 (a biofilm-forming strain). The data are presented as mean ± standard deviation (n = 2), P < 0.05 statistically significant; *P < 0.05, **P < 0.005, ***P < 0.001, ***P < 0.0001; one-way ANOVA using Dunnett’s correction for multiple comparisons.

Biofilm formation was observed in both clinical and environmental strains (Figure 2). 37°C for 24–72 h was the determined condition for strong biofilm formation. At 72 h, there was formation of mature biofilms with characteristic strong adherence to the walls of the tube and plate after crystal violet staining. The strains were categorized as negative, weak, moderate or strong based on the biofilm-forming index standard. The majority (66%) of the strains displayed a strong biofilm phenotype (Figure 2). Both clinical and environmental *K. pneumoniae*, *P. aeruginosa*. and *Enterobacter* sp. formed strong biofilms while environmental *Citrobacter* sp., clinical *A. baumannii* were weak biofilm formers. Clinical *Citrobacter* sp. and *E. coli* were moderate formers. In total, 63% of the environmental strains displayed a stronger biofilm phenotype compared to the clinical strains (37%).

**FIGURE 3:**
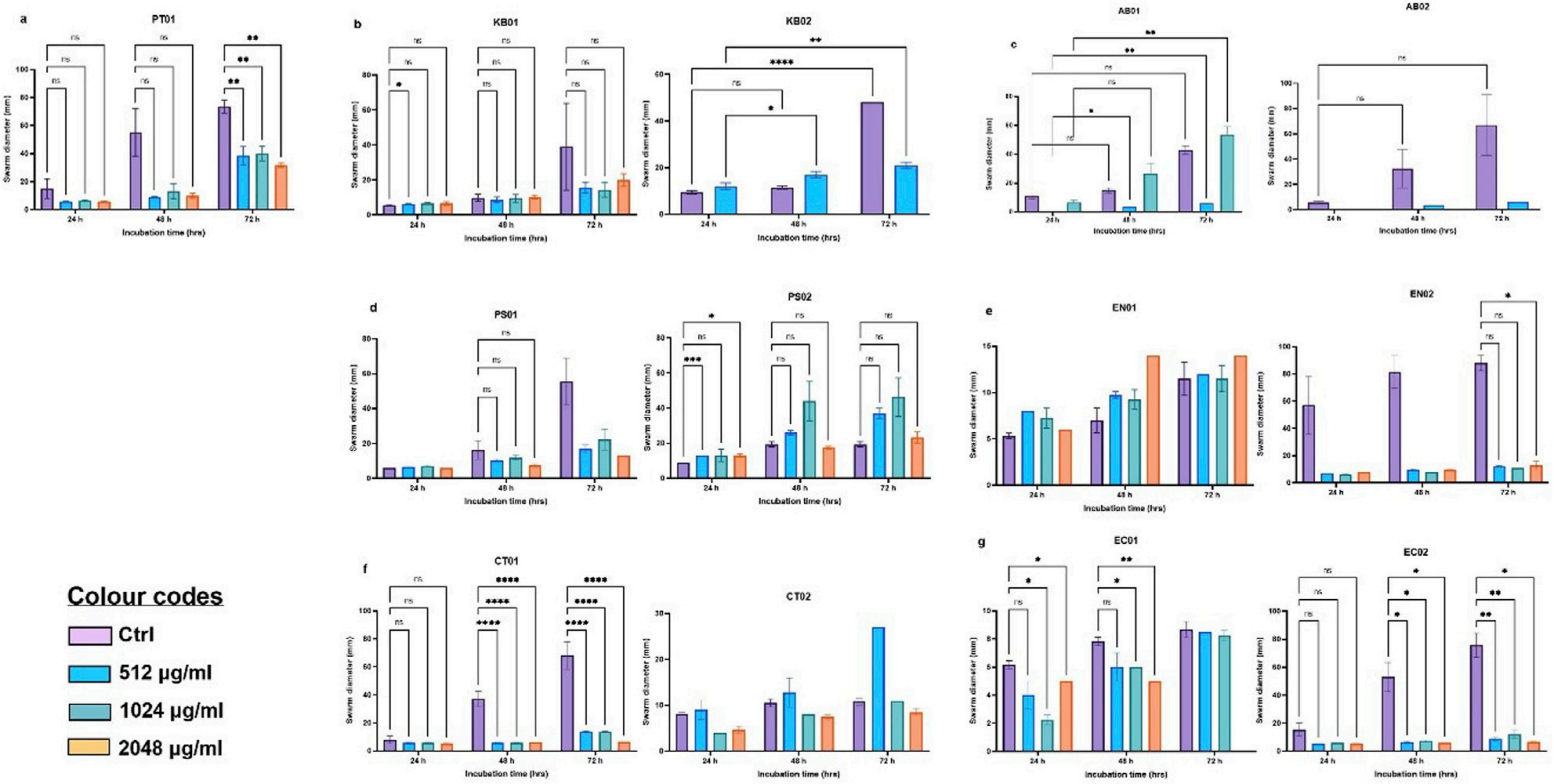
Swarm profile of clinical and environmental strains under PMB stress **(A)** PT01; **(B)** *K. pneumoniae* (KB01, KB02); **(C)** *A. baumannii* (AB01, AB02); **(D)** *P. aeruginosa* (PS01, PS02); **(E)** *Enterobacter* sp. (EN01, EN02); **(F)** *Citrobacter* sp. (CT01, CT02); **(G)** *E. coli* (EC01, EC02). The data are presented as mean ± standard deviation (n = 2) P < 0.05 statistically significant, *P < 0.05, **P < 0.005; two-way ANOVA with Dunnett’s correction for multiple comparisons was used.

Surface-motility, particularly swarming was determined by measuring the mean diameter of the swarms formed at 37°C (for 24, 48 and 72 h). Strains were described as non-motile with <5 mm diameter, 5 – 20 mm as intermediate and >20 mm as strong swarmers. For the majority of the strains, swarming was gradual while others showed a sharp increase in diameter after 24 h (Figure 3). Eight of the strains showed robust motility with diameters above 20 mm after 48 h (KB01, KB02, AB01, AB02, PS01, EN02, CT01, EC02). Clinical *P. aeruginosa* (PS02), *Citrobacter* sp. (CT02), environmental *E. coli* (EC01) and *Enterobacter* sp. (EN01) were intermediate swarmers. At 512 μg/mL, 1,024 μg/mL and 2048 μg/mL PMB, swarming was significantly reduced relative to the respective wild types (Figure 3). The majority of the strains exhibited an intermediate motility phenotype (5 – 20 mm) in the presence of PMB with strains of *K. pneumoniae* (KB02) and *A. baumannii* (AB01 and AB02) being inhibited at 1,024 and 2048 μg/mL, respectively (Figures 3B,C).

### Strains formed biofilms in the presence of polymyxin B

**FIGURE 4:**
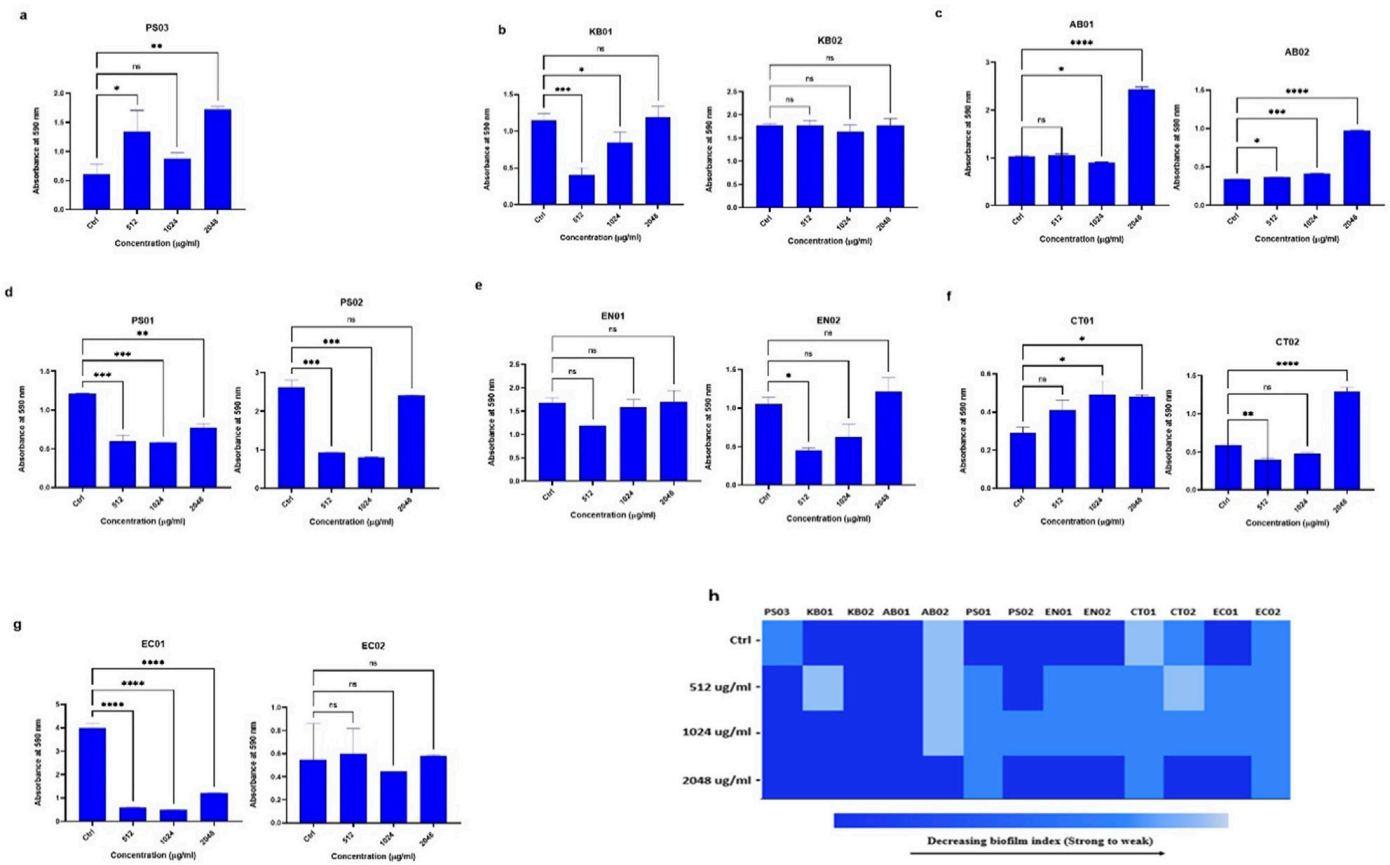
Assessment of biofilm inhibition in the presence of PMB **(A)** *Pseudomonas* sp. (PS03) **(B)** *K. pneumoniae* (KB01, KB02); **(C)** *A. baumannii* (AB01, AB02); **(D)** *P. aeruginosa* (PS01, PS02); **(E)** environmental and clinical strain of *Enterobacter* sp. (EN01, EN02); **(F)** *Citrobacter* sp. (CT01, CT02); **(G)** *E. coli* (EC01, EC02). **(H)** Heat map of biofilm index after PMB inhibition assay. Color codes represent weak, moderate and strong biofilms. The data are presented as mean ± standard deviation (n = 2) P < 0.05 statistically significant; *P < 0.05, **P < 0.009, ***P < 0.001; one-way ANOVA using Dunnett’s correction for multiple comparisons.

At 512 μg/mL, 1,024 μg/mL and 2048 μg/mL PMB treatment, strains formed biofilms (Figure 4). There was no complete inhibition and eradication of biofilm, but the degree of biofilm formed relative to the controls (without PMB) varied. There was an increase in biofilm biomass with increasing antibiotic concentration. For the determination of biofilm inhibition (Figure 4B), environmental *K. pneumoniae* (KB02) assumed a weak biofilm phenotype at 512 μg/mL relative to its strong phenotype in the absence of PMB. At 1,024 μg/mL and 2048 μg/mL, it formed strong biofilms. However, clinical *K. pneumoniae* formed strong biofilms at all PMB concentrations (Figure 4B), indicating no biofilm inhibition. The clinical *A. baumannii* strain formed a weak biofilm at 512–1024 μg/mL with a strong phenotype at 2048 μg/mL, while the environmental strain maintained a strong biofilm phenotype irrespective of PMB concentrations (Figure 4C). Biofilm formation in environmental *P. aeruginosa* was moderate at all antibiotic concentrations with the clinical strain displaying moderate biofilm formation at 1,024 μg/mL (Figure 4D). Environmental *Enterobacter* sp. was observed as a strong biofilm former under antibiotic pressure while the clinical strain displayed a moderate phenotype at 512 μg/mL and 1,024 μg/mL but a strong one at 2048 μg/mL (Figure 4E). The environmental *Citrobacter* sp. strain formed a weak biofilm in the absence of antibiotics, but showed a moderate biofilm phenotype at the three antibiotic concentrations (Figure 4F), while the clinical strain was observed as weak, moderate and strong at 512 μg/mL, 1,024 μg/mL and 2048 μg/mL respectively. In *E. coli*, the environmental strain (EC01) displayed a moderate phenotype at 512 μg/mL and 1,024 μg/mL, while the clinical strain (EC02) was observed as moderate at all the antibiotic concentrations (Figure 4G). Overall, the selected antibiotic concentrations did not inhibit biofilm formation, although some strains exhibited reduced biofilm (Figure 4H).

### High concentrations of polymyxin B could not eradicate preformed biofilms

**FIGURE 5:**
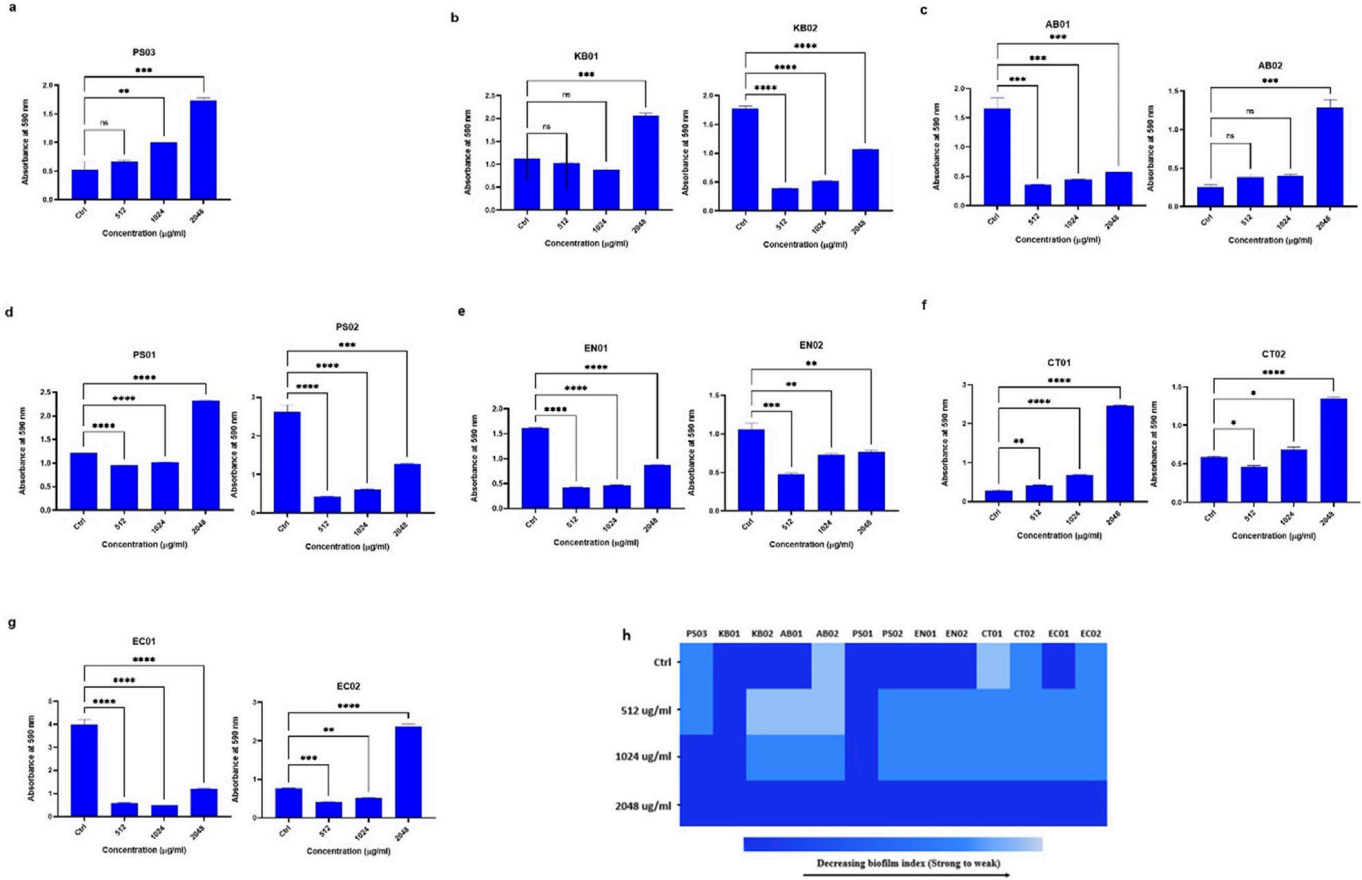
Assessment of biofilm eradication in the presence of PMB **(A)** *Pseudomonas* sp. **(B)** *K. pneumoniae* (KB01, KB02); **(C)** *A. baumannii* (AB01, AB02); **(D)** *P. aeruginosa* (PS01, PS02); **(E)** *Enterobacter* sp. (EN01, EN02); **(F)** *Citrobacter* sp. (CT01, CT02); **(G)** *E. coli* (EC01, EC02). **(H)** Heat map of biofilm index after PMB eradication assay. Color codes represent weak, moderate and strong biofilms. The data are presented as mean ± standard deviation (n = 2) P < 0.05 statistically significant; ns (P > 0.05); *P < 0.05, **P < 0.009, ***P < 0.001; one-way ANOVA using Dunnett’s correction for multiple comparisons.

To determine the concentration of antibiotic needed to reduce preformed biofilm (Figure 5A), strains were challenged with PMB at MIC concentrations (512 μg/mL) (Figures 5A-G). In general, the biofilm index of all the strains at 2048 μg/mL was strong, indicating that the antibiotic concentration did not eradicate the preformed biofilm (Figures 5B-G). At 1,024 μg/mL, the biofilm index ranged between moderate and strong (Figure 5C). At 512 μg/mL, there was a reduction in preformed biofilm from a strong index to a moderate index for the majority of the strains with clinical *K. pneumoniae* and *A. baumannii* (AB01 and AB02) assuming a weak phenotype (Figure 5B). In general, the degree of biofilm eradicated at 512 μg/mL was greater than at 1,024 μg/mL and 2048 μg/mL (Figure 5H).

### Bacterial strains were viable after biofilm inhibition and eradication assay

**FIGURE 6:**
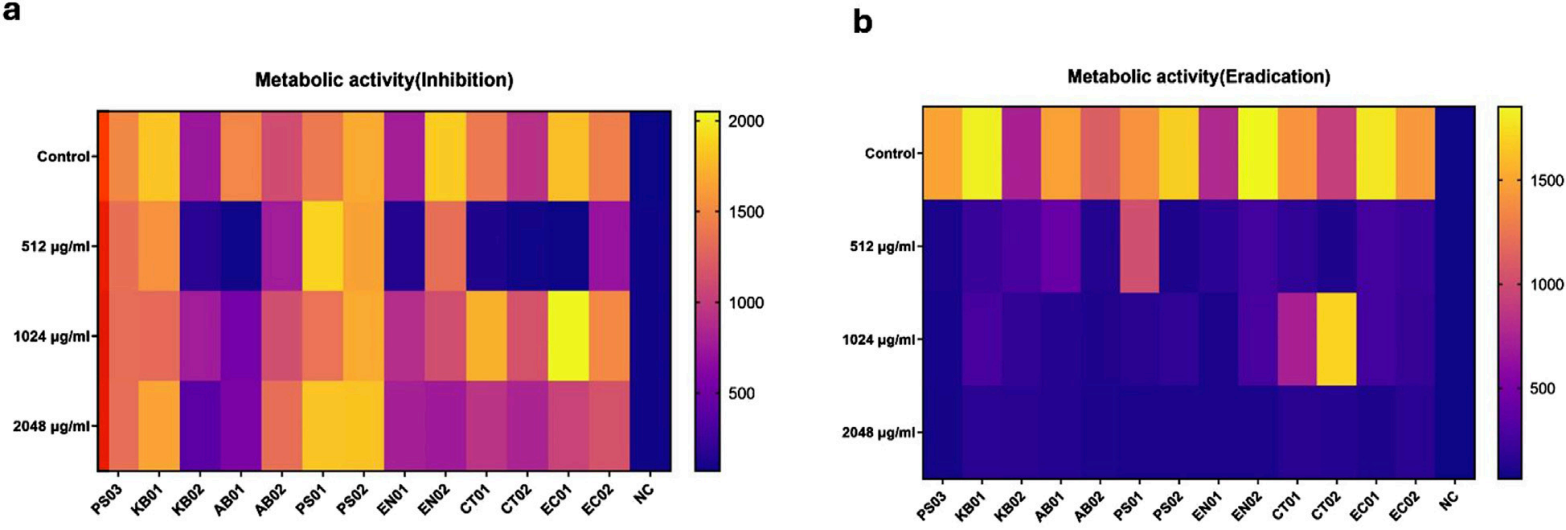
Metabolic activity of strains after PMB treatment at 24 h. MBIC and MBEC metabolic heatmap. The horizontal bar represents the degree of RFU measurement. Relative to controls (upper rows), each row panel represents treatment with PMB at 512 μg/mL, 1,024 μg/mL and 2048 μg/mL respectively. Two-way ANOVA with Dunnett’s correction for multiple comparisons was used to compare metabolic activity between strains and controls. For the inhibition assay, there was a significant difference in metabolic activity of strains relative to NC (p < 0.0028) compared to PS03 (p > 0.1). For the eradication assay, no significant difference in the metabolic activity of strains relative to PS03 (p > 0.05) and NC (p > 0.05). P < 0.05 statistically significant; ns (P > 0.05); *P < 0.05, **P < 0.009, ***P < 0.001.

The viability of the strains was inferred by employing a resazurin metabolic assay after inhibition and eradication assays. Strains were observed as metabolically active 24 h after incubation for the inhibition assay (Figure 6A) with some strains displaying metabolic activity 48 h after eradication (Supplementary – 48 h Metabolic Eradication). Relative fluorescence units (RFU) ranged from 1,500–2000, indicating a difference in strain reduction ability and viability of the cell population (MBIC). Some strains (KB01, PS02, EN02, and EC01) reduced resazurin more rapidly than others (KB02, EN01, CT02, and EC01); however, reduction was observed in all strains after biofilm formation and indication of strain viability (Figure 6A). For inhibition (MBIC), reduction/viability was higher at 1,024 μg/mL and 2048 μg/mL compared to 512 μg/mL for the majority of strains. There was no significant difference in the metabolic activity of the strains relative to the PS03, but there was a significant difference in cell viability after the inhibition assay relative to the negative control (NC). In the eradication assay (Figure 6B), the majority of the strains displayed low RFU levels at the 3 antibiotic concentrations. Although the reduction was low in MBEC (Figure 6B), relative to the negative control, a reduction was observed in all strains mainly at 48 h (Supplementary – 48 h Metabolic Eradication), indicating a degree of viability after antibiotic treatment.

## Discussion

This study characterized Gram-negative KAPE pathogens, *Citrobacter* spp. and *E. coli* from Ghanaian hospital environments and explored their phenotypic profiles (biofilm and swarm motility) under polymyxin B pressure. The clinical and environmental isolates displayed average (80%–85%) levels of AMR to the tested antibiotics. This raises concern as clinical isolates are expected to be more resistant than environmental isolates due to antibiotic exposure during treatment [39]. The resistance patterns exhibited by environmental isolates suggest that these strains have either acquired mobile genetic elements in the environment or have a tendency to adapt to environmental stress factors [40]. The MIC to polymyxin B was 512 μg/mL; seven times higher than the CLSI standard for AMR for *A. baumannii, P. aeruginosa* and *Enterobacter* spp. The presence of polymyxin-resistant strains in the hospital environment increases the risk of treatment failure during infection. The resistance pattern displayed to both conventional antibiotics and polymyxin B confers a multidrug-resistant phenotype to the strains. This relatively high level of resistance observed is particularly worrying in Ghana and adds to the growing reports of AMR observed globally [3, 41].

Surface-motility and biofilm formation can be described as complex adaptations associated with adaptive multidrug resistance, bacterial persistence, and virulence [21]. The colonization ability of the strains was analyzed *in vitro* by studying the biofilm-motility interplay. In this study, all strains exhibited surface movement on the semi-solid agar from the point of inoculation after 24 h. The degree of swarming was conditional, strain-specific and dependent on the ability of bacterial strains to generate and maintain moisture on the agar surface as described by Carabarin-Lima et al., 2016 and Lai et al., 2009 [26, 42]. *Proteus* sp. exhibited increasing swarming with increasing incubation times at 37°C. The majority of the strains displayed swarming to a lesser degree relative to the control (PT01); however, clinical strains of *A. baumannii*, *Enterobacter* sp., and *E. coli* and the environmental strain of *Citrobacter* sp. showed higher swarming ability after 48 h. Although some strains are classified as non-motile (*Klebsiella* sp. & *Acinetobacter* sp.), they have a tendency to display motility, which is evident as swarming on agar plates [42, 43]. It is possible that the halos observed on the agar plates for *Acinetobacter* sp. could employ twitching or surface-associated motility [44–46] and flagella-mediated motility for *Klebsiella* under certain defined conditions [42]. This is particularly important as motility is also characterized as a host invasion and evasion strategy. Motility regulation is often coupled to the expression of virulence determinants including the ability to invade diverse cell types, leading to persistent infections [44, 45]. The clinical relevance of surface-motility is particularly observed in acute infections, as it allows for rapid colonization and establishment of infection [46]. The incidence of HAIs is enhanced by the pathogenic tendencies of opportunistic KAPE pathogens.

The strains also exhibited swarming under polymyxin B pressure. Previous studies have reported higher AMR during bacterial swarming in *P. aeruginosa*, *S. marcescens* [26] and *S. enterica* [47]. As swarming is a multicellular coordinated behavior, cell density confers a protective layer to withstand exposure to lethal concentrations of antibiotics [48, 49]. Death in a subpopulation of bacterial cells could enhance adaptive resistance in the surviving population [50] leading to reduced swarm diameter but persistent swarming; this is evident in the intermediate phenotype observed. Although both clinical and environmental strains displayed intermediate swarming and polymyxin B tolerance at 512, 1,024, 2048 μg/mL, surface-motility for *Klebsiella* (KB02) and *A. baumannii* (AB01 and AB02) was inhibited at higher concentrations, an indication of cell death. Overall, the presence of polymyxin B reduced the swarming ability of the strains but did not result in total cell death for the majority of strains relative to the *Proteus* sp. control. This suggests that surface-motility, particularly swarming could be a mechanism employed by bacteria to resist lethal concentrations of antibiotics, resulting in AMR and persistent infections in immunocompromised individuals in hospital settings [21].

We explored the ability of the strains to form biofilms following polymyxin B stress. Biofilm-associated bacteria can cause chronic infections that persist, unlike their planktonic counterparts, causing acute infections [51]. Biofilm formation can be suppressed through inhibition of the planktonic population, preventing initial adhesion, and removing established biofilm [52]. All strains were biofilm formers with some exhibiting higher degrees of biofilm biomass. Based on the biomass index, the majority of the strains (66%) were strong formers in the absence of antibiotics. Biofilm can be described as a stress-induced response to environmental factors such as exposure to antimicrobials to enable bacterial persistence during infection. The MIC of 512 μg/mL established for the planktonic cells did not inhibit biofilm formation even at 2048 μg/ml. A study of sub-MIC concentrations of antibiotics was reported to induce biofilm formation by a factor of 2 in *P. aeruginosa* [48], indicating that higher concentrations would induce even stronger biofilm phenotypes. In addition, Černohorská & Votava, [48] showed that strains of *P. aeruginosa*, *K. pneumoniae, A. baumannii* and *E. coli* exhibited enhanced survival and resistance in a biofilm population relative to the planktonic cells.

As a result of the difficulty in eradicating biofilms, some studies suggest increasing the antibiotic concentrations [52, 53]. In the eradication assay, the degree of biofilm was reduced at 512 μg/mL relative to the untreated control. Eradication did not result in complete clearance of the biofilm formed but in a moderation of the biofilm index, similar to reports that polymyxin B led to a reduction of preformed biofilm in *S. aureus*, *E. coli* [54] and *Pseudomonas* sp. [55]. Clearance of preformed biofilm cells is difficult, hence a multistep combination antibiotic treatment is required to efficiently reduce biofilm biomass in KAPE pathogens [56]. There was, however, strong biofilm formation in all strains at 2048 μg/mL, indicating the role of higher antibiotic concentration in inducing stronger biofilms and resistance [52]. When comparing the swarm and biofilm profiles, all strains that exhibited surface-motility formed biofilms at higher concentrations of polymyxin B. The robust swarming nature and the biofilm profile exhibited by the strains indicate a positive correlation between biofilm formation and swarming motility in our strains. Microbial biofilms and swarms pose a significant challenge in the hospital environment, as they influence antibiotic resistance phenotypes and enhance persistent infections in that setting.

To determine the viability of biofilm cells, a resazurin assay was adopted, in which metabolically active cells reduce blue non-fluorescent resazurin to pink and highly fluorescent resorufin [53]. The wild type reduction of the majority of the strains was above 1500 RFU relative to below 200 RFU for the negative control, indicating the presence of a viable number of active bacterial cells after biofilm development. During inhibition, fluorescence readings were above 500 RFU at 1,024 and 2048 μg/mL. Some studies have reported different metabolizing abilities of cells, such as *S. aureus,* which rapidly reduces resazurin compared to *P. aeruginosa* and *B. cenocepacia* [57]. The removal of the stress factor resulted in strains assuming a metabolically active phenotype; however, at 512 μg/mL, the majority of the strains displayed low metabolic activity at 24 h post-stress conditions. The reduction in biofilm at 512 μg/mL during inhibition corresponds to the reduction in cell viability observed at 512 μg/mL in the resazurin assay. Comparing the concentration of PMB at 512 and 1,024 μg/mL, sub-MIC concentrations of PMB could further reduce bacterial viability in a biofilm environment compared to increasing antibiotic concentration. In the eradication assay, a reduction of 500 RFU was recorded for the majority of the strains, which could indicate lower numbers of viable cells or dormancy after stress. Studies have reported low nutrient availability to bacterial cells in the deeper layers of a biofilm, leading to dormancy in this state with reversal of dormancy after stress removal [32]. Strains within a biofilm inhibition and eradication assay could be characterized as metabolically active and slow-growing strains, respectively. Metabolically active cells enhance biofilm formation and induce resistance phenotypes through the expression of diverse enzymes required for strain survival [58]. Additionally, preformed biofilms are characterized by slow-growing cells with reduced antibiotic efficacy due to biofilm biomass and reduced metabolic activity [58-61].

## Conclusion

The clinical and environmental strains displayed appreciably similar growth and AMR patterns indicating that the strains could be described as multidrug resistant. The ability to form biofilm and display robust surface-motility, particularly under polymyxin B pressure indicates a greater ability to tolerate, resist and survive under high antibiotic pressure. Since polymyxin B did not significantly inhibit or reduce the degree of biofilm formed, the tendency for increased pathogenesis and virulence of infections during host colonization is possible. The presence of viable cells particularly during biofilm inhibition indicates a growing tolerance to antibiotics and therefore a need for guided treatment options. The challenge of resistance coupled with the ability of strains to exhibit phenotypes corresponding to characteristics that enhance infection persistence in the hospital environment is challenging. These characteristics are particularly concerning, as environmental isolates exhibit similar phenotypes to clinical isolates. This poses a challenge to treatment outcomes and the subsequent spread of AMR in a closed environment such as the ICU. More worryingly, these strains are present in Ghanaian hospital environments, implying the need to intensify research into the mechanisms of AMR and explore possible therapeutic interventions.

## Data Availability

The datasets presented in the study are deposited in Harvard Dataverse as supplementary files and can be found here - https://doi.org/10.7910/DVN/JJUUKN.
